# Arctigenin Inhibits Lung Metastasis of Colorectal Cancer by Regulating Cell Viability and Metastatic Phenotypes

**DOI:** 10.3390/molecules21091135

**Published:** 2016-08-27

**Authors:** Yo-Han Han, Ji-Ye Kee, Dae-Seung Kim, Jeong-geon Mun, Mi-Young Jeong, Sang-Hyun Park, Byung-Min Choi, Sung-Joo Park, Hyun-Jung Kim, Jae-Young Um, Seung-Heon Hong

**Affiliations:** 1 Department of Oriental Pharmacy, College of Pharmacy, Wonkwang-Oriental Medicines Research Institute, Wonkwang University, 460 Iksandae-ro, Iksan, Jeonbuk 54538, Korea; dygks1867@hanmail.net (Y.-H.H.); keejy8627@naver.com (J.-Y.K.); dr0marvin@naver.com (D.-S.K.); wjdrjs92@hanmail.net (J.-g.M.); 2 Center for Metabolic Function Regulation, Wonkwang University, Iksan, Jeonbuk 54538, Korea; jeongmy1@hanmail.net; 3 Isotope Sciences Lab, Korea Atomic Energy Research Institute, Jeongeup, Jeonbuk 54538, Korea; parksh@kaeri.re.kr; 4 Department of Biochemistry, School of Medicine, Wonkwang University, Iksan, Jeonbuk 54538, Korea; bmchoi@wonkwang.ac.kr; 5 Department of Herbology, College of Oriental Medicine, Wonkwang University, Iksan, Jeonbuk 54538, Korea; parksj0822@hanmail.net; 6 Department of Herb Science, Dong-eui institute of technology, 54 Yangji-ro, Busanjin-gu, Busan 47230, Korea; herb@dit.ac.kr; 7 Department of Pharmacology, College of Korean Medicine, Institute of Korean Medicine, Kyung Hee University, 26, Kyungheedae-ro, Dongdaemun-gu, Seoul 02447, Korea

**Keywords:** Arctigenin, colorectal cancer, apoptosis, lung metastasis

## Abstract

Arctigenin (ARC) has been shown to have an anti-cancer effect in various cell types and tissues. However, there have been no studies concerning metastatic colorectal cancer (CRC). In this study, we investigated the anti-metastatic properties of ARC on colorectal metastasis and present a potential candidate drug. ARC induced cell cycle arrest and apoptosis in CT26 cells through the intrinsic apoptotic pathway via MAPKs signaling. In several metastatic phenotypes, ARC controlled epithelial-mesenchymal transition (EMT) through increasing the expression of epithelial marker E-cadherin and decreasing the expressions of mesenchymal markers; N-cadherin, vimentin, β-catenin, and Snail. Moreover, ARC inhibited migration and invasion through reducing of matrix metalloproteinase-2 (MMP-2) and MMP-9 expressions. In an experimental metastasis model, ARC significantly inhibited lung metastasis of CT26 cells. Taken together, our study demonstrates the inhibitory effects of ARC on colorectal metastasis.

## 1. Introduction

Arctigenin (ARC), a lignan-derived compound, is isolated from various kinds of plants such as *Arctium lappa* Linné (Arctii Fructus) and *Forsythia suspensa* *Vahl* (Forsythia Fruit) [1,2]. ARC has several pharmacological activities, including anti-tumor, anti-inflammatory, anti-oxidant, and anti-diabetic activities [3]. Recent studies have shown that ARC suppresses the production of nitric oxide and inducible nitric oxide synthase as well as p38 mitogen-activated protein kinase (MAPK) and nuclear transcription factor-kappa B (NF-κB) pathways, which contribute to cancer cell growth and survival [4,5]. However, it remains unclear whether ARC has inhibitory effects on colorectal metastasis.

Colorectal cancer (CRC) is the third most diagnosed cancer and second leading cause of cancer-related mortality. In the United States, about 1.6 million new cancer cases were diagnosed in the year 2013. Among them, 142,000 cases are diagnosed with CRC, and 50,830 patients out of 142,000 cases are dying of CRC. The early stage of noninvasive adenomas can be cured by surgical excision, but there are few effective therapies for patients suffering from advanced forms of CRC and the survival rate is also very low [6,7].

A balance between stimulators and inhibitors of cell proliferation controls the cell cycle and a deregulation of the cell cycle leads to an uncontrolled proliferation of cancer cells [8]. Cell cycle decontrol is a feature of cancer cells. So, cell cycle arrest, which is associated with inhibition of cell proliferation, is a crucial target of anti-cancer treatment strategy. Down-regulation of cyclin-dependent kinase subunits (CDKs) induced cell cycle arrest and, therefore, could be an important anti-cancer activity [9,10]. 

Apoptosis serves as a crucial process for blocking metastasis, because apoptosis prevents metastatic dissemination through the elimination of circulating tumor cells. Pro- and anti-apoptotic Bcl-2 family members interact in apoptotic process. Bcl-2 and Bcl-xL, the anti-apoptotic proteins, can antagonize pro-apoptotic proteins, such as Bax [11], and they induce the activation of caspases. Therefore, regulating apoptosis-related proteins is a potential therapeutic possibility and these proteins are key targets for the development of anti-cancer drugs [12,13].

EMT is involved in malignant tumor progression and metastasis. EMT is a cellular process during which epithelial cells gain mesenchymal features and lose their cell-to-cell contacts. EMT triggers detachment of cancer cells from the primary cancer organ and triggers invasion into lymphatic or blood vessels through the loss of intercellular junctions [14,15]. Several EMT-related markers, including epithelial and mesenchymal genes expression, are modulated during EMT process. Snail is a major EMT switch transcription factor that increases N-cadherin, β-catenin, and vimentin and decreases E-cadherin expression [16].

Matrix metalloproteinases (MMPs) have been considered as major factors in accelerating metastasis. MMPs are extracellular proteases and zinc-binding endopeptidases which are related to the degradation of extracellular matrix (ECM) and affect a crucial role in metastasis such as cancer cell growth, migration and invasion. MMPs are divided into 2 groups: soluble MMPs and transmembrane-type MMPs. MMP-2 and MMP-9 are important members of soluble MMPs and play important roles in cancer development. These molecules are considered as gelatinases related to the degradation of type IV collagen. As type IV collagen is the major component of the basement membrane, MMP-2 and MMP-9 have crucial roles in the early stages of cancer invasion and metastasis [17,18].

In this study, we investigate the anti-metastatic effects of ARC using metastatic colon cancer cell lines and an experimental animal metastasis model. 

## 2. Results

### 2.1. ARC Induces Cell Death of Colon Cancer Cells

**Figure 1:**
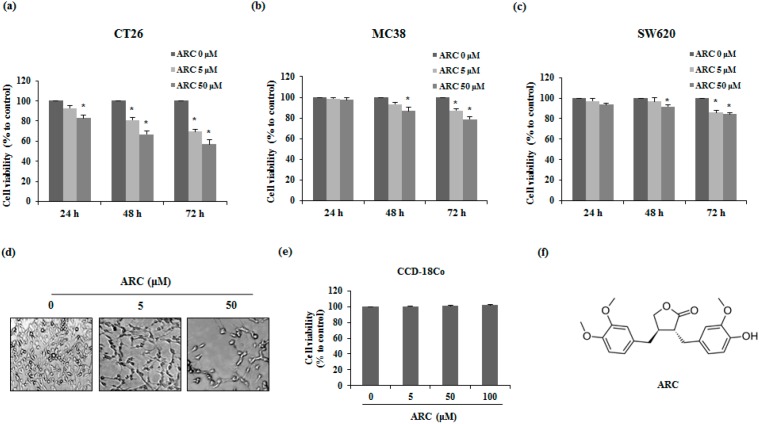
ARC decreases cell proliferation of metastatic colon cancer cells. (**a**–**c**) Cell viability of ARC-treated CT26 (**a**), MC38 (**b**), and SW620 (**c**) cells; (**d**) the morphology of ARC-treated CT26 cells for 72 h. Photographs were acquired by microscopy; (**e**) cell viability of ARC-treated CCD-18Co cells, which are normal colon cell. The cells were seeded at a density of 3 × 10^3^ cells/well in 96-well microplates and treated with ARC for 72 h. After incubation at 37 °C, cell viability was examined using the MTS assay; and (**f**) chemical structure of ARC. Results are expressed as the mean ± SD. * *p* < 0.05.

To evaluate whether ARC has cytotoxicity on metastatic colon cancer cells, CT26, MC38, and SW620 cells were used. The 3-(4,5-dimethylthiazol-2-yl)-5-(3-carboxymethoxyphenyl)–2(4-sulfophenyl)-2*H*-tetrazolium, inner salt (MTS) assay was used to elucidate the cytotoxicity effect of ARC on the various colon cancer cell lines. As is shown in Figure 1a, ARC decreased the cell viability of CT26 cells and the apoptotic morphological changes were detected by microscopic observation (Figure 1d). Moreover, proliferations of MC38 and SW620 cells were decreased by ARC treatment in a dose- and time-dependent manner (Figure 1b,c). To confirm the impacts of ARC on the viability of normal colon cell lines, we used CCD-18Co cells. As shown in Figure 1e, ARC did not affect normal colon cell lines at experimental concentrations. In our previous study, 100 μM treatment of ARC significantly reduced the viability of 3T3-L1 pre-adipocytes, whereas 50 μM ARC did not show any cytotoxicity [19]. Therefore, we performed further experiments with 50 μM ARC as a high concentration.

### 2.2. ARC Increases Cell Cycle Arrest in G2/M1 Phase and Induces Apoptosis in Colon Cancer Cells

**Figure 2:**
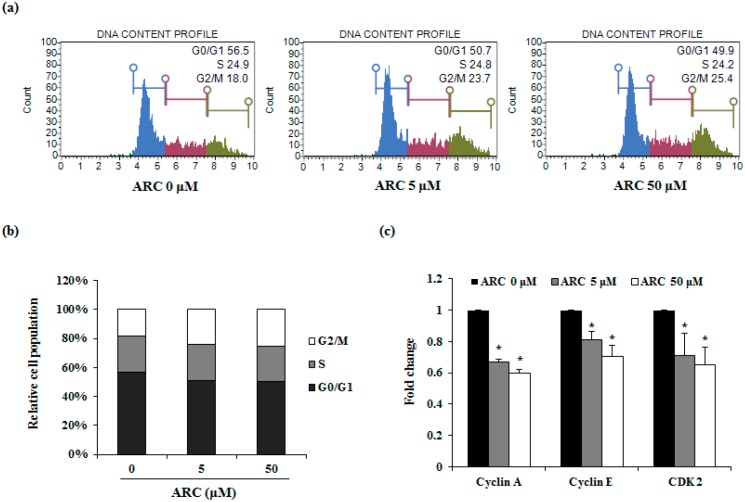
ARC induces cell death via cell cycle arrest. (**a**) Cell cycle analysis of CT26 cells after ARC treatment for 24 h; (**b**) the percentage of cell cycle phases; and (**c**) mRNA expression levels of cyclin A, cyclin E and CDK2. CT26 cells were treated with ARC for 24 h. Results are expressed as the mean ± SD. * *p* < 0.05.

To investigate whether the growth inhibitory effect of ARC on CT26 cells was partly due to cell cycle change, flow cytometry was used. CT26 cells were treated with various concentrations of ARC for 24 h and the DNA content of the cells was measured. After various concentrations of ARC were treated, the G2/M1 phase of CT26 cells was blocked (Figure 2a,b). To further confirm that the increasing percentage of cells in G2/M1 was induced by ARC, we performed real-time RT-PCR to detect cyclin A, cyclin E, and CDK 2 expressions. ARC inhibited the mRNA expression of cyclin A, cyclin E, and CDK 2 (Figure 2c). These results indicate that ARC-mediated cell cycle arrest in CT26 cells was associated with a decrease of expression of cyclin A, cyclin E, and CDK 2.

### 2.3. ARC Induces Apoptosis via MAPKs in Colon Cancer Cells

**Figure 3:**
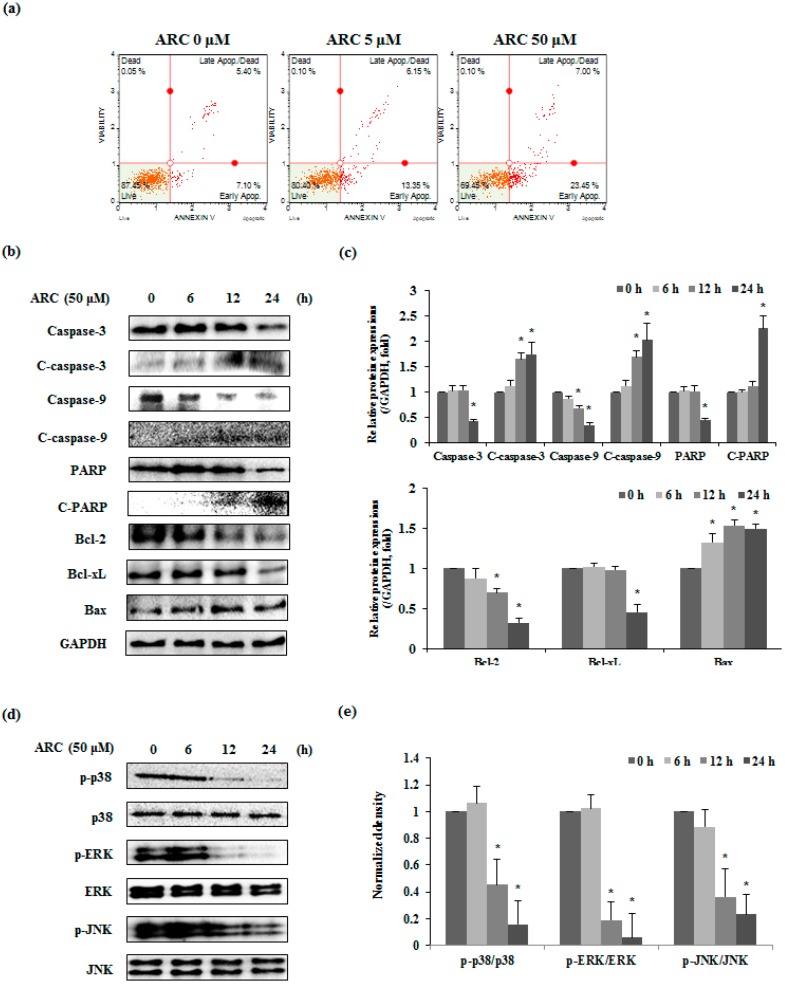
ARC induces cell death via apoptosis. (**a**) CT26 cells were incubated with the indicated concentrations of ARC for 48 h and stained with Annexin V and PI; (**b**,**c**) CT26 cells were treated with ARC for 0–24 h and confirmed by Western blotting for caspase-3, -9, PARP, Bcl-2, Bcl-xL, and Bax, respectively. The band intensities were measured by densitometry and presented as the relative ratio to GAPDH; (**d**,**e**) CT26 cells were treated with ARC (50 μM) for 0–24 h and phosphorylation of p38, ERK, and JNK were detected by Western blotting. The band intensities were measured by densitometry and presented as the relative ratio. Results are expressed as the mean ± SD. * *p* < 0.05.

To confirm whether the ARC-induced cell death is apoptosis or not, CT26 cells were treated with ARC (0, 5, or 50 μM) for 48 h and Annexin V assay was performed. As shown in Figure 3a, ARC increased both early (lower right of Figure 3a) and late apoptosis (upper right of Figure 3a) in CT26 cells. To further confirm the mechanism of apoptosis by ARC, apoptosis related proteins were investigated by Western blot analysis. Exposure of CT26 cells to ARC (50 μM) for 0–24 h caused a cleavage of caspases-3 and -9. In addition, ARC caused time dependent decreases in the Bcl-2, Bcl-xL, and poly ADP-ribose polymerase (PARP), whereas the expression level of Bax increased in ARC-treated CT26 cells (Figure 3b). Since MAPK’s signaling pathway is involved in apoptosis of tumor cells, we supposed that these results were related to MAPK’s, including p38, ERK, and JNK. As shown in Figure 3d and e, ARC treatment significantly inhibited phosphorylation of p38, ERK, and JNK.

### 2.4. ARC Inhibits the Migration and Invasion Ability and Regulates EMT Transition of CT26 Cells

**Figure 4:**
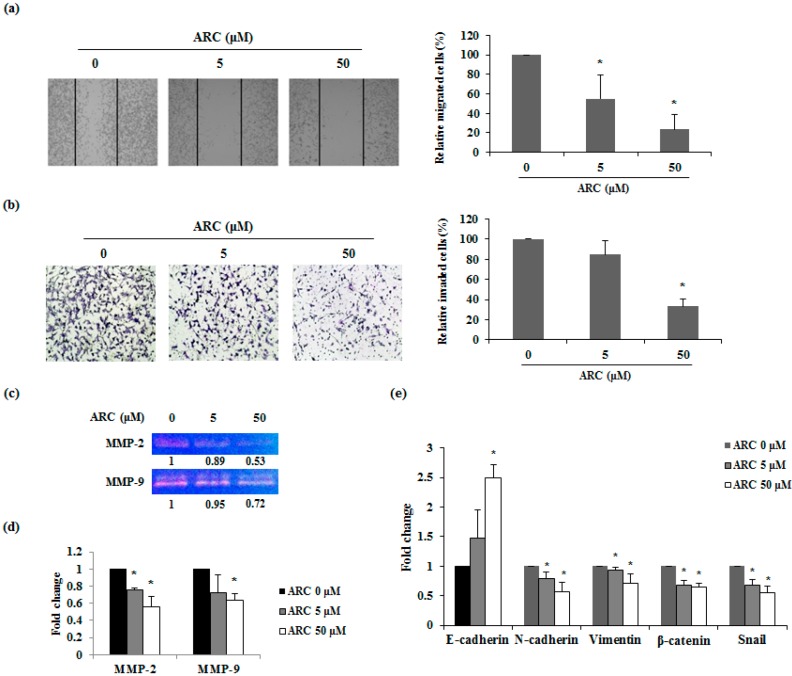
ARC reduces migration, invasion and mRNA expression of EMT transition markers. (**a**) Wound healing assay; and (**b**) invasion assay. Images were taken using a microscope (100× magnification); (**c**) Activities of MMP-2 and -9 in ARC-treated CT26 cells were determined by gelatin zymography; (**d**) the mRNA expression levels of MMP-2, -9; and (**e**) EMT transition markers were analyzed by real-time RT-PCR after ARC treatment. Results are expressed as the mean ± SD. * *p* < 0.05.

Migration and invasion are the fundamental features of metastasis after EMT. Therefore, wound healing assay was performed to confirm whether ARC inhibits the migration of CT26 cells. Cell movements were observed 24 h after ARC treatment. In the presence of ARC, cell migration was decreased in a concentration-dependent manner. Control group cells migrated toward the scratched site, whereas the migration of ARC-treated cells decreased in a dose-dependent manner (Figure 4a). The invasion ability of the CT26 cells was also measured by the matrigel invasion assay. As is shown in Figure 4b, ARC induced significant decreases in the invasion of CT26 cells. Next, gelatin zymography was conducted to analyze the activity of MMP-2 and MMP-9. As shown in Figure 4c, ARC decreases the activity of MMP-2 and MMP-9 in CT26 cells. Additionally, to determine the molecular mechanism of the anti-invasive ability of ARC, the mRNA expression of MMP-2 and MMP-9 were measured. The results showed that mRNA expression levels of MMP-2 and MMP-9 was down-regulated by ARC treatment in a dose-dependent manner (Figure 4d). To confirm whether ARC controls the expression of EMT transition markers, which are closely related to metastatic properties, the mRNA expression of EMT transition factors was determined. ARC treatment significantly increased the expression of the epithelial marker E-cadherin in CT26 cells. However, the mesenchymal markers N-cadherin, vimentin, β-catenin, and Snail were significantly decreased in ARC-treated cells (Figure 4e). These results indicated that ARC decreased the EMT and metastatic abilities through inhibition of MMP-2 and MMP-9 expressions and activities in CRC cells.

### 2.5. ARC Ameliorates Lung Metastasis of CT26 Cells in an Experimental Metastasis Model

**Figure 5:**
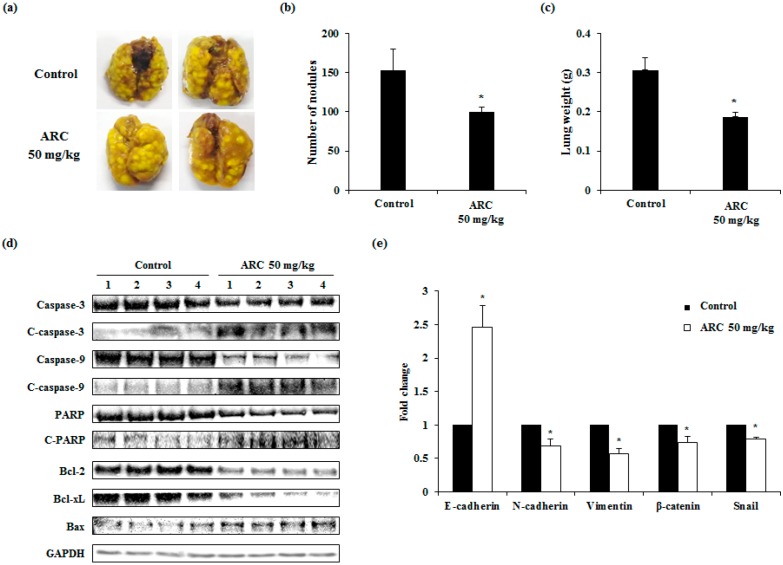
ARC reduces colorectal lung metastasis. BALB/c mice were intravenously transplanted with 1 × 10^5^ cells of CT26 cancer cells into the tail vein (i.v.). Then, the mice were divided into two groups (*n* = 8). ARC (50 mg/kg/day) was administered by oral gavage once a day until sacrifice. (**a**) Lungs were excised and stained with Bouin’s solution to compare the pattern of pulmonary tumor nodule formation among the experimental groups; (**b**) the number of tumor nodules and (**c**) lung weight are expressed as the mean ± SD (*n* = 4); (**d**) the protein levels of apoptotic proteins were detected in lung tissues (*n* = 4); and (**e**) the mRNA expression levels of EMT transition markers were measured in lung tissues (*n* = 4). Results are expressed as the mean ± SD. * *p* < 0.05.

Our study also investigated whether ARC could ameliorate lung metastasis of CRC cells in mice. As is shown in Figure 5a–c, the numbers of pulmonary tumor nodules were decreased by the oral administration of ARC (50 mg/kg/day) for two weeks, by about 30%. Compared to the control group, ARC-treated mice showed significantly lower tumor formations. We then confirmed the apoptotic proteins and EMT markers in lung tissues. ARC administration induced apoptosis and inhibited the EMT transition in lung tissues (Figure 5d,e). These results indicate that ARC could decrease the aggravation of colon cancer metastasis.

## 3. Discussion

ARC is a lignin-type compound contained in various plants, including *Arctium lappa* L. Several studies have demonstrated that ARC has anti-oxidant, anti-viral, and anti-inflammatory effects. In particular, ARC shows anti-tumor effects through inducing necrosis caused by inhibiting mitochondria respiration in A549 lung carcinoma cells and promoting cell cycle arrest or apoptosis in gastric cancer cells and breast cancer cells [20,21]. This study aimed to elucidate the anti-metastatic effects of ARC, which still remain unclear, using metastatic CRC cells and an experimental animal model.

Malignant CRC is related to high rates of metastasis and mortality. Although surgery remains the most crucial treatment for CRC, multiple approaches are needed to prevent and improve of metastasis. For good prognosis, it is important that the inhibition of cancer cell proliferation is performed in the early stages [22]. In a previous study, it has been reported that ARC has a growth inhibitory effect in the human colorectal cancer cell line SW480. The IC50 of ARC was 42.5 μM in SW480 cells after 48 h treatment [23]. Since we focused on anti-metastatic effect of ARC, cytotoxicity of ARC was measured using human metastatic CRC cell line SW620. Although the anti-proliferative effect of ARC (0–50 μM) in SW620 cells was weak, ARC significantly decreased proliferation of SW620 cells in this study. Moreover, we confirmed non-cytotoxicity of ARC until 100 μM concentration in normal colon cell line CCD-18Co cells (Figure 1e). However, 100 μM of ARC showed inhibition of cell growth in 3T3-L1 adipocytes [19]. Therefore, we conducted subsequent experiments using 0–50 μM of ARC. As is shown in Figure 1, our results demonstrate the anti-proliferation effect of ARC on metastatic CRC cells, including CT26, MC38 and SW620. 

Induction of cell cycle arrest and apoptosis is one of the major anti-cancer activities [24]. Our results show that ARC induced cell cycle arrest by increasing the percentage of cells in G2/M phase in CT26 cells (Figure 2a,b). The expression of cyclin A is closely related to G2/M phase in cancer cells [25]. Cyclin E/CDK2 complex also controls G2/M phase. These factors ultimately promote G2/M transition and finally allow them to enter mitosis [26]. Therefore, regulation of these factors’ expression is important for the anti-proliferation of cancer cells. In this study, expression of cyclin A, cyclin E, and CDK2 were reduced by ARC treatment (Figure 2c). Since ARC also induced apoptosis (Figure 3), as well as the cell cycle arrest effect of ARC being weak on CT26 cells, ARC may partially decrease cell proliferation by inducing cell cycle arrest through the inhibition of cyclins and CDK expressions in CT26 cells.

Apoptosis is controlled by two main pathways, which are the extrinsic receptor-mediated pathway and the intrinsic mitochondria-dependent pathway [27]. The intrinsic pathway was controlled by Bcl-2 family proteins, which regulate mitochondria-dependent apoptosis. Bcl-2 and Bcl-xL are anti-apoptotic proteins, which prevent the emission of cytochrome c from mitochondria. By contrast, Bax is a pro-apoptotic protein that promotes the release of cytochrome c. Cytochrome c from the mitochondria activates caspase-9. Eventually, activated caspase-9 subsequently induces caspase-3 and PARP cleavage [28]. In our results, ARC inhibited cell proliferation by inducing the apoptosis of CT26 cells (Figure 3a). The cleavage of caspase-3, -9, and PARP was increased by ARC treatment (Figure 3b). Moreover, ARC treatment inhibited the expressions of Bcl-2 and Bcl-xL, whereas Bax expression was increased (Figure 3b). The MAPKs signaling pathway is involved with various cellular processes such as migration, proliferation, apoptosis, and cell cycle arrest [29]. This pathway is also related with both extrinsic and intrinsic apoptotic pathways. In a previous study, ARC treatment decreased the phosphorylation of p38, ERK and JNK (Figure 3c). Thus, ARC is believed to induce apoptosis via the MAPK’s signaling pathway and caspases/Bcl-2 family proteins.

MMPs are closely associated with the migration and invasion ability of cancer cells. The role of MMP-2 and MMP-9 in metastasis is to degrade proteins in the extracellular matrix, and eventually affect cancer progression and invasion [30]. A low concentration of ARC inhibited movement and invasion of CT26 cells (Figure 4a,b). In addition, activity and expression of MMP-2 and MMP-9 were significantly decreased by ARC treatment. These results suggest that ARC could inhibit the migration and invasion ability by decreasing the activity and expression of MMP-2 and MMP-9 in CT26 cells (Figure 4c,d).

EMT, which is related to the loss of cell-to-cell contacts through obtaining mesenchymal features, is crucial for metastasis [31]. In this study, we examined whether ARC could regulate the expression of EMT-related genes. ARC up-regulated the expression of the epithelial marker, E-cadherin, and down-regulated the expression of mesenchymal markers, including N-cadherin, vimentin, β-catenin, and Snail (Figure 4e). These results indicated that ARC could have anti-metastatic potential through the inhibition of the EMT process.

We also used an experimental metastasis in vivo model. This study showed that ARC significantly decreased the number of tumor nodules and lung weight (Figure 5a–c). Moreover, the expressions of apoptotic proteins and EMT related genes were ameliorated by ARC treatment (Figure 5d,e).

## 4. Experimental Section

### 4.1. Antibodies and Reagents

ARC was purchased from Sigma Chemicals Co. (St. Louis, MO, USA). Anti-PARP, caspase-3 and caspase-9 antibodies were purchased from Cell Signaling Technology, Inc. (Danvers, MA, USA). GAPDH and Bcl-2 antibodies were purchased from Santa Cruz Biotechnology, Inc. (Santa Cruz, CA, USA). Bcl-xL and Bax antibodies were purchased from Bioworld Technology (Louis Park, MN, USA).

### 4.2. Cell Culture

The mouse colon carcinoma cell lines colon 26 (CT26), colon 38 (MC38), and CCD-18Co were maintained in Dulbecco’s Modified Eagle’s Medium (DMEM; Gibco BRL, Grand Island, NY, USA). The human colon carcinoma cell line SW620 was maintained in RPMI1640 (Gibco BRL, Grand Island, NY, USA) These media were supplemented with 10% heat-inactivated fetal bovine serum (FBS), 100 units/mL penicillin, and 100 μg/mL streptomycin at 37 °C in a 5% CO_2_ incubator. 

### 4.3. Animals

BALB/c female mice (5 weeks, 19–20 g) were purchased from Da-Mool Science (Daejeon, Korea). The animals were housed in a laminar air-flow room maintained at a temperature of 22 ± 1 °C and a reactive humidity of 55% ± 1%. ARC was dissolved in 0.3% CMC water and was administered by oral gavage once a day until sacrifice. The research was conducted in accordance with internationally accepted principles for laboratory animal use and care, as stated in Wonkwang University guidelines (WKU15-103).

### 4.4. Assays of Cell Viability

Cell viability was quantified using the cell proliferation reagent MTS Kit (Promega Corporation, WI, USA), as recommended by the manufacturer. Cells were seeded in 96-well microplates at 3 × 10^3^ cells/well and an ARC containing medium was added to the wells. After 24–72 h incubation, the medium was changed with MTS solution, and absorbance was measured at 490 nm.

### 4.5. Annexin V Assay

Annexin V assay was carried out using the FITC Annexin V Apoptosis Detection Kit I (BD Biosciences, San Diego, CA, USA). In brief, harvested cells were washed twice with a cold phosphate-buffered saline (PBS) and the cells were resuspended in a 1× Annexin V binding buffer (1 × 10^6^ cells/mL). Then, 100 μL of the solution (1 × 10^5^ cells) was transferred to a 5 mL culture tube and labeled with 3 µL titrated FITC Annexin V and Propidium Iodide (PI) staining solution. After the cells were vortexed, they were incubated for 15 min at room temperature in the dark. The volume was then made up to 500 μL and analyzed with the FACS Calibur system (BD Biosciences, San Diego, CA, USA).

### 4.6. Western Blot Analysis

CT26 cells (1 × 10^6^ cells/well) were incubated with various concentrations of ARC. Stimulated cells were rinsed with PBS and then lysed in lysis buffer (iNtRon Biotech, Seoul, Korea) for 1 h. Cell lysates were centrifuged for 10 min and the quantity of protein was evaluated by using a bicinchoninic acid (BCA) protein assay. The supernatant was mixed with a 2× sample buffer for SDS-PAGE gel electrophoresis and transferred to an immobilon-P-nylon membrane (Millipore, Bedford, MA, USA). The membranes were blocked with 5% skim milk for 1 h 30 min and incubated for 3 h with primary antibodies. The antibodies were detected using horseradish peroxidase-conjugated anti-rabbit, anti-mouse, and immunoglobulin G (Dako, Glostrup, Denmark) and blots were detected using the ECL system (Santa Cruz, CA, USA).

### 4.7. Cell Cycle Analysis

Cell cycle distribution was measured using the Muse Cell Cycle Kit (Millipore, MA, USA) according to the manufacturer’s instructions. Briefly, CT26 cells (1 × 10^6^ cells) were plated in six-well plates and incubated with various concentrations of ARC for 24 h. Cells were harvested and fixed with 70% ice cold ethanol at −20 °C for at least 3 h. After washing with PBS, cell pellets were resuspended in 200 μL of Cell Cycle Reagent and incubated for 30 min at room temperature in the dark. Cells were analyzed by Muse Cell Analyzer and the cell cycle phase distribution was quantified using Muse analysis software (Millipore, MA, USA).

### 4.8. Real-Time RT-PCR

Total RNA was isolated from CT26 cells using QIAzol lysis reagent (QIAGEN sciences, MD, USA) according to the manufacturer’s directions. First-strand cDNA was prepared from an RNA template (2 μg) using an oligo (dT)18 primer and a Power cDNA Synthesis Kit (iNtRon Biotech). Reverse transcription was performed at 42 °C for 50 min and then at 70 °C for 15 min. Real-time quantitative RT-PCR was performed using a Power SYBR^®^ Green PCR Master Mix and StepOne Plus^TM^ Real-time PCR Systems (Applied Biosystems, Foster City, CA, USA). All data were normalized to GAPDH. The primer sequences are summarized in Table 1.

### 4.9. Wound Healing Assay

CT26 cells were seeded in a six-well plate (2 × 10^5^ cells/well) to form a monolayer. Using a 200 μL pipette tip, a scratch of ~1 mm width was made in triplicate. Detached cells were removed, and the scratches were monitored at regular intervals over the course of 0–24 h under serum-free conditions containing ARC at indicated concentrations. Images were acquired under a phase contrast microscope (Leica, Wetzlar, Germany).

### 4.10. Invasion Assay

The invasion ability of cancer cells was estimated using a BD BioCoat GFR Matrigel invasion chamber (BD Biosciences, San Diego, CA, USA). CT26 cells (5 × 10^4^ cells) were suspended in serum-free DMEM with ARC and added to the upper part of the transwell chamber. For the invasion assay, the lower part of the transwell was filled with 10% FBS in DMEM as a chemoattractant. After 24 h of incubation, the membrane inserts were washed twice with PBS and fixed with 3.7% paraformaldehyde in PBS for 5 min. After being washed twice with PBS, the fixed cells were treated with 100% methanol for 20 min and stained with Giemsa for 15 min. The inner sides of the chamber were wiped with a cotton swab. The membrane inserts were dried and observed under a microscope.

### 4.11. Gelatin Zymography

Cells were seeded in six-well plates (3 × 10^5^ cells/well) and incubated for overnight. The cells were maintained in serum-free medium for 12 h prior to treatment with ARC for 24 h. Conditioned media from the cells were diluted 1:1 in zymography sample buffer, and subjected to electrophoresis on a 8% SDS-PAGE gel containing 1% gelatin. The gels were renatured by incubation with renaturing buffer (pH 7.5, 2.5% Triton X-100 in D.W) for 30 min at room temperature and incubated in developing buffer (50 mM Tris-HCl pH 7.5, 10 mM CaCl_2_, and 150 mM NaCl) at 37 °C overnight. The gelatinolytic activity of MMPs was visualized by staining the gels with Coomassie blue R-250 solution for 30 min.

### 4.12. Experimental Lung Metastasis Model

For experimental lung metastasis, mice were divided into 3 groups (*n* = 8). BALB/c mice were inoculated with CT26 (1 × 10^5^ cells) in 200 μL of PBS via the lateral tail vein (i.v.), and each mouse was administrated with an oral injection of ARC (50 mg/kg/day) every day for 14 days. Control group mice were administered with the same volume of water. The mice were sacrificed 14 days after treatment and the lungs were removed and fixed in Bouin’s solution (Sigma, St. Louis, MO, USA). Lung weight and the number of tumor colonies in the lungs were measured to evaluate tumor metastasis.

### 4.13. Statistical Analyses

Results were expressed as mean ± standard deviation (SD) of independent experiments, and statistical analysis was performed using Student’s *t*-test. All statistical analyses were performed using SPSS statistical analysis software version 11.5 (SPSS Inc., Chicago, IL, USA). Values with *p* < 0.05 were considered to indicate statistical significance.

## 5. Conclusions

Our results first demonstrated the anti-cancer and anti-metastatic effects of ARC in colon cancer cells in vitro and in vivo. ARC induced cell death through apoptosis and cell cycle change, which was found to be associated with the MAPKs pathways. Moreover, ARC inhibited EMT, migration, and invasion both in vivo and in vitro. Based on our findings, ARC is a potential therapeutic agent for colorectal lung metastasis therapy.

## Figures and Tables

**Table 1:** Sequences for real-time RT-PCR primers.

Gene	Forward (5′-3′)	Reverse (5′-3′)
MMP-2	CCCCATGAAGCCTTGTTTACC	TTGTAGGAGGTGCCCTGGAA
MMP-9	AGACCAAGGGTACAGCCTGTTC	GGCACGCTGGAATGATCTAAG
E-cadherin	AATGGCGGCAATGCAATCCCAAGA	TGCCACAGACCGATTGTGGAGATA
N-cadherin	TGGAGAACCCCATTGACATT	TGATCCCTCAGGAACTGTCC
Snail	TCCAAACCCACTCGGATGTGAAGA	TTGGTGCTTGTGGAGCAAGGACAT
β-catenin	ACTGCTGGGACTCTG	TGATGGCGTAGAACAG
Vimentin	CGGAAAGTGGAATCCTTGCA	CACATCGATCTGGACATGCTG
GAPDH	GACATGCCGCCTGGAGAAAC	AGCCCAGGATGCCCTTTAGT
